# The tumor suppressor adenomatous polyposis coli regulates T lymphocyte migration

**DOI:** 10.1126/sciadv.abl5942

**Published:** 2022-04-13

**Authors:** Marta Mastrogiovanni, Pablo Vargas, Thierry Rose, Céline Cuche, Elric Esposito, Marie Juzans, Hélène Laude, Amandine Schneider, Mathilde Bernard, Sophie Goyard, Charlotte Renaudat, Marie-Noëlle Ungeheuer, Jérôme Delon, Andrés Alcover, Vincenzo Di Bartolo

**Affiliations:** 1 Institut Pasteur, Université de Paris, INSERM-U1224, Unité Biologie Cellulaire des Lymphocytes, Ligue Nationale Contre le Cancer, Équipe Labellisée Ligue 2018, F-75015 Paris, France.; 2 Sorbonne Université, Collège Doctoral, F-75005 Paris, France.; 3 Institut Curie, PSL Research University, CNRS, UMR 144, F-75005 Paris, France.; 4 Institut Pierre-Gilles de Gennes, PSL Research University, F-75005 Paris, France.; 5 Institut Pasteur, Université de Paris, UTechS BioImagerie Photonique, F-75015 Paris, France.; 6 Institut Pasteur, Université de Paris, ICAReB, F-75015 Paris, France.; 7 Université de Paris, Institut Cochin, Inserm, CNRS, F-75014 Paris, France.

## Abstract

Adenomatous polyposis coli (APC) is a tumor suppressor whose mutations underlie familial adenomatous polyposis (FAP) and colorectal cancer. Although its role in intestinal epithelial cells is well characterized, APC importance in T cell biology is ill defined. APC regulates cytoskeleton organization, cell polarity, and migration in various cell types. Here, we address whether APC plays a role in T lymphocyte migration. Using a series of cell biology tools, we unveiled that T cells from FAP patients carrying APC mutations display impaired adhesion and motility in constrained environments. We further dissected the cellular mechanisms underpinning these defects in APC-depleted CEM T cell line that recapitulate the phenotype observed in FAP T cells. We found that APC affects T cell motility by modulating integrin-dependent adhesion and cytoskeleton reorganization. Hence, APC mutations in FAP patients not only drive intestinal neoplasms but also impair T cell migration, potentially contributing to inefficient antitumor immunity.

## Teaser

APC regulates T cell migration by controlling integrin-dependent adhesion, cytoskeleton organization and membrane protrusions.

## INTRODUCTION

Familial adenomatous polyposis (FAP) is an autosomal dominant inherited disease that often results from germline mutations in the adenomatous polyposis coli (APC) gene, a tumor suppressor and polarity regulator. APC protein regulates the Wnt/β-catenin signaling pathway as a component of the β-catenin destruction complex. This pathway is crucial for intestinal epithelium homeostasis. APC mutations alter epithelial cell differentiation, proliferation, polarization, and migration, thus disrupting tissue architecture (*1*). FAP patients carrying APC gene mutations develop numerous precancerous colorectal polyps growing from the age of 10 to 12 years, with high risk of cancer by the age of 40 (*2*). Thus, APC alterations are prerequisite for progression toward malignancy.

APC is a polarity regulator also necessary for polarized cell migration (*3*). Through its N-terminal and C-terminal domains, APC interacts with both actin and microtubule regulatory molecules controlling microtubule network organization and dynamics and actin polymerization (*4*–*6*). Last, APC controls the organization of intermediate filaments (*7*).

The key role of immunity in human colorectal carcinoma was underscored by the correlation between the type and frequency of infiltrating T lymphocytes and favorable patient prognosis (*8*, *9*). Once activated in lymph nodes, lymphocytes go through bloodstream recirculation and adhesion-dependent trans-endothelial migration to finally invade tumor tissues, where they execute their effector functions to eliminate tumor cells. Lymphocyte migration through endothelium and tissues occurs in response to chemokine cues and relies on the coordination of acto-myosin and microtubule cytoskeleton dynamics and of integrin-mediated adhesion (*6*, *10*, *11*). T cell migration involves the formation of a lamellipodium at the leading edge and a uropod at the trailing edge. Integrins, as LFA-1 (α_L_β_2_) and VLA-4 (α_4_β_1_), concentrate at various cellular areas, ensuring firm adhesion by binding to ICAM-1 and VCAM-1 on vascular endothelial cells and to fibronectin in extracellular matrix. Various adhesion molecules (i.e., CD44, ICAM-1, ICAM-2, and ICAM-3) concentrate at the uropod, supporting cell-cell interactions. Integrins and uropod adhesion molecules are linked to the cortical actin cytoskeleton via talin and ezrin and moesin proteins, respectively (*12*–*14*). Cell polarity complexes orchestrate the cytoskeletal cross-talk needed for directional migration (*6*).

Our recent work highlighted the association of APC with microtubules in T cells (*15*, *16*) and showed that APC defects impair cytoskeleton organization at immunological synapses of CD4 and CD8 T cells, compromising differentiation and anti-inflammatory function of intestinal T regulatory cells (*15*) and cytotoxic activity of CD8 T cells (*16*). This revealed new roles of APC in T cell effector functions crucial for antitumor immunity.

Here, we unveiled that T lymphocytes from FAP patients carrying APC mutations show defective chemokine-induced migration through micropores or human umbilical vein endothelial cell (HUVEC) monolayers. Defects of FAP patients’ cells were maintained when migrating through chemokine-free, fibronectin-coated microchannels, indicating a switch from lamellipodium- to bleb-driven migration. Moreover, VLA-4 integrin expression was diminished in FAP patients’ T cells, likely contributing to their defective adhesion to VCAM-1– or fibronectin-coated surfaces. Last, studying an APC-silenced T cell line as a complementary model of APC defects, we revealed that APC-silenced cells displayed less structured lamellipodia and impaired filopodia formation. Together, we show a concomitant impact of APC defects on adhesion and cytoskeleton organization, resulting in impaired T cell migration.

Therefore, APC mutations in FAP patients may weaken both intestinal epithelium homeostasis and antitumor immune surveillance, which relies on the ability of the immune cells to migrate to and through transformed tissues.

## RESULTS

### T lymphocytes from patients carrying APC mutations display impaired migration

**Fig. 1.**
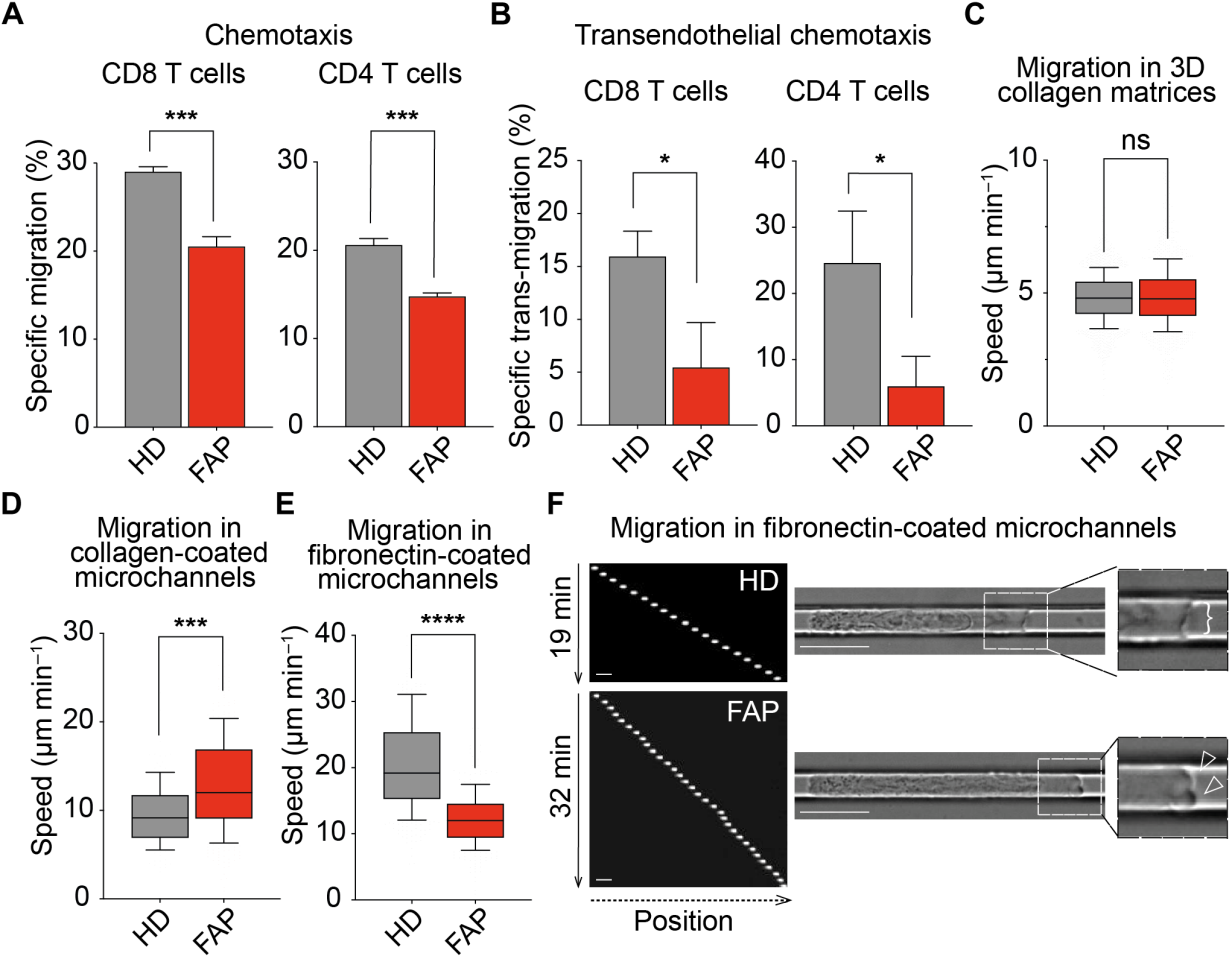
T cells from FAP patients present migration defects. (**A**) Transmigration through transwell filters was assessed for T cells from FAP patients (FAP) and matched healthy donors (HD). Bars represent mean + SEM (six individual pairs) of specific migration of CD8 and CD4 T cells. Statistical differences were calculated by paired *t* test. ***P < 0.001. (**B** to **F**) T cells from FAP patients and matched HDs were reactivated after thawing. (B) Bar graphs represent mean + SEM (three individual pairs) of specific transwell migration of CD8 and CD4 T cells through HUVEC monolayers. Statistical differences were calculated by paired *t* test. *P < 0.05. (C to F) CD8 T cells were sorted after thawing and were assessed for their migration in 3D collagen gels (C) or in microchannels (D to F). Boxes illustrate 10th to 90th percentiles of values, and whiskers represent the range of values. Statistical differences were calculated by Mann-Whitney unpaired test. ***P < 0.001 and ****P < 0.0001; ns, not significant. (C) CD8 T cell migration in 3D collagen matrices was recorded by time-lapse video microscopy. Mean speed of migrating cells from four individual pairs is shown. (D to F) T cell migration through collagen type I–coated (D) or fibronectin-coated (E and F) microchannels was assessed for CD8 T cells from three and five matched individuals, respectively. (F) Left panel shows sequential images of Hoechst-labeled CD8 T cells from a representative individual pair (20× objective). Scale bars, 10 μm. Right panels show video microscopy frames (63× objective) (movie S1). Insets zoom in the front edge’s areas of migrating cells displaying cell lamellipodium (bracket) or lobe-shaped protrusions or blebs (empty arrowheads).

In nonlymphoid cells, as astrocytes, APC is at the crossroads of cytoskeleton and mechanical forces necessary to promote polarized cell migration (*3*). We hypothesized that APC is involved in T lymphocyte migration that may be impaired in cells from FAP patients carrying APC mutations. Therefore, we assessed the ability of 5-day activated T cells from FAP patients versus age- and sex-matched healthy donors to migrate through transwell filters in response to CXCL12 chemokine. Chemokine-induced migration of both CD4 and CD8 T cells from FAP patients was significantly impaired as compared to their matched controls (Fig. 1A). T cell growth and size were similar when T cells were activated ex vivo (fig. S1), suggesting that no apparent defects in immune cell differentiation and turnover occurred in these patients.

To investigate the potential consequences of APC defects in a more physiological setting, we analyzed T cell chemotaxis through HUVEC monolayers. For these experiments, we made use of T cells from three FAP patients and their matched healthy volunteers that had been conserved frozen and set back in culture. Cells from FAP patients displayed a diminished capacity to migrate through HUVEC monolayers (Fig. 1B). Together, these data show that FAP T cells have a decreased capacity to perform chemotaxis toward CXCL12 through micropores or endothelial barriers.

We further studied FAP T cell migration at the single-cell level in the absence of chemokines to address whether this motility impairment is due to an intrinsic migration defect. We concentrated in the study of the CD8 T cell population since it was the most enriched upon activation. First, we measured migration of T cells in three-dimensional (3D) collagen matrices, in which leukocytes rely mostly on cell contractility to move (*17*, *18*). In this system, motility of CD8 T cells from FAP patients was not impaired with respect to matched healthy subjects (Fig. 1C and fig. S2, B and C).

We next used microchannels in which cells migrate in a straight line, providing a simplified assay to study T cell locomotion (*19*, *20*). Cells from FAP patients showed a significant increase in their migration when moving in collagen-coated microchannels (Fig. 1D). Together, these data indicate that FAP CD8 T cells do not have intrinsic defects of their migration capacity in collagen-coated substrates. It is worth noting that the expression of integrin α_1_ and α_2_ subunits, which ensure collagen binding when pairing with β_1_, was rather low and comparable between healthy donors and FAP patients (see fig. S2D).

Since T cell migration can be influenced by their adhesion to the extracellular matrix, we performed migration experiments in microchannels coated with fibronectin, which binds to VLA-4 at the T cell surface. Notably, migration speed of FAP CD8 T cells was significantly diminished under this condition (Fig. 1, E and F). In addition, these cells appeared more elongated and progressed emitting multiple bleb-like protrusions at the leading edge, while control cells displayed a larger front lamellipodium, indicating different modes of migration on fibronectin-coated surfaces (Fig. 1F and movie S1).

Together, these data provide the first evidence that APC contributes to regulate T cell migration in confined microenvironments, as often found in tissues. The impact of APC depends on the composition of the extracellular matrix, indicating an adhesion-dependent phenomenon.

### T lymphocytes from patients carrying APC mutations display impaired VLA-4–mediated adhesion

**Fig. 2.**
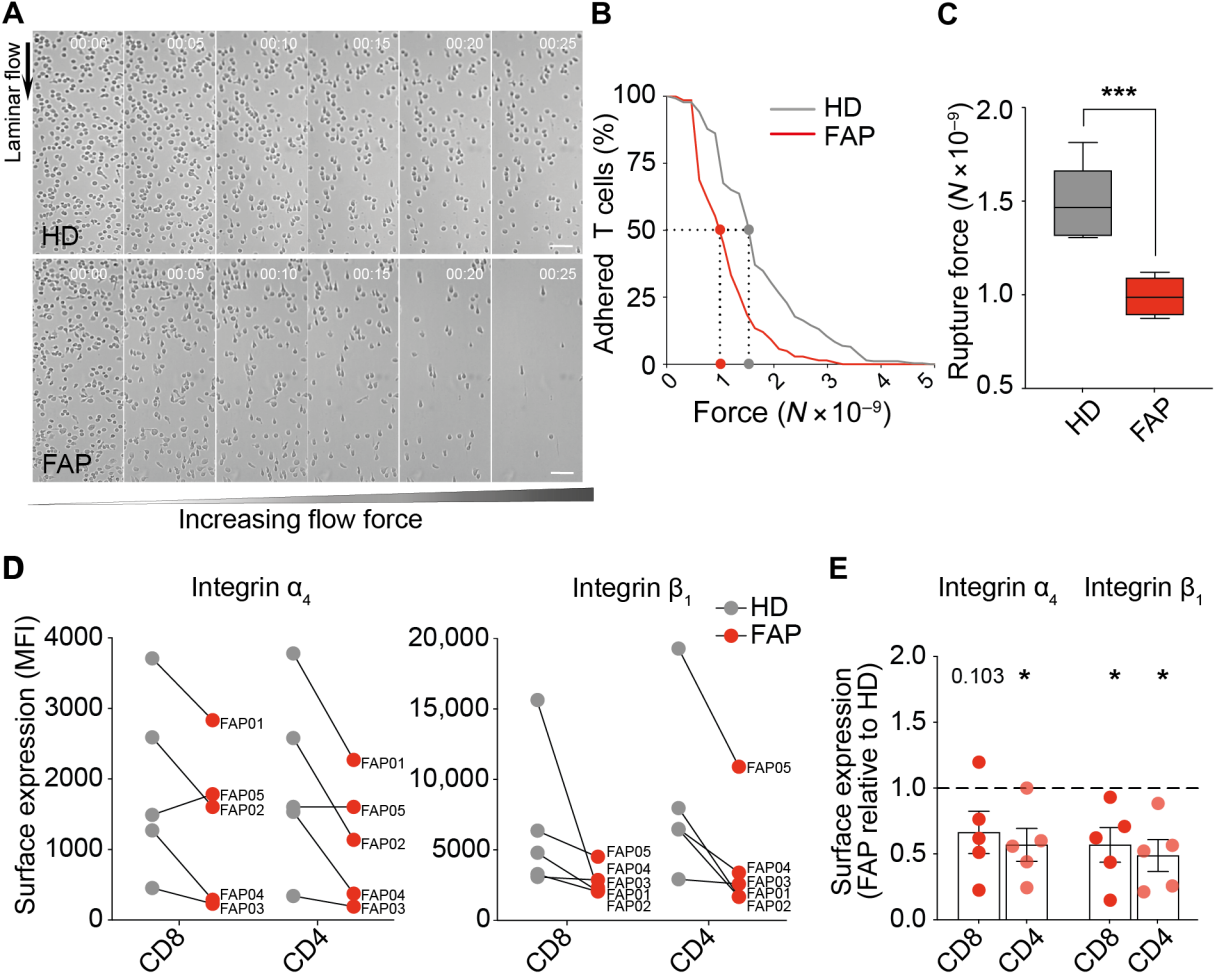
T lymphocytes from FAP patients display altered integrin-mediated adhesion. Adhesion strength of T cells from FAP patients (FAP) and their matched healthy donors (HD) to VCAM-1 + CXCL12–coated surface was assessed by shearing flow (A to C), and surface expression of the α_4_β_1_/VLA-4 integrin (VCAM-1 ligand) in these cells was analyzed by flow cytometry (D to E). (**A** to **C**) CD8 T cells were seeded in a laminar flow chamber and submitted to increasing flow rates. Sequential images of detaching cells from a representative pair of HD and FAP are shown in (A) (movie S2). Flow’s direction is indicated (10× objective). Scale bars, 50 μm. (B) The number of adherent cells per frame was plotted for this representative individual pair as a function of the force. Dotted lines mark the rupture force necessary for detaching 50% of the cells from the substrate. (C) Boxes represent the rupture force of adhesion to VCAM-1 + CXCL12–coated surfaces of T cells from seven FAP patients and matched HDs. Statistical differences were calculated by Mann-Whitney unpaired test. ***P < 0.001. (**D** and **E**) Surface expression of α_4_ and β_1_ integrin subunits was measured by flow cytometry in five subjects’ pairs. (D) Linked dots show the median fluorescence intensity (MFI) of VLA-4 subunits for each FAP patient and its matched HD. (E) VLA-4 median fluorescence intensity of each FAP patient was normalized to the one of his matched HD. Each dot represents one FAP patient, and the dashed line shows the reference value of HD (normalized to 1). Statistical differences were calculated by one-sample two-tailed *t* test. *P* values are indicated or replaced by * when *P* < 0.05.

The importance of adhesion to fibronectin to discriminate control from APC mutant cells prompted us to analyze T cell adhesion forces to the fibronectin-interacting integrin VLA-4, using shear flow microchambers. To prevent heterogeneity of fibronectin coating, we used the cellular ligand for VLA-4, VCAM-1, in the presence of CXCL12 chemokine that enhances integrin-mediated adhesion (*21*). When increasing shear force was applied to bound CD8 T cells, FAP patient’s T cells detached at lower force than those from healthy subjects (Fig. 2, A to C, and movie S2), indicating that APC mutations result in decreased T cell adhesion to VCAM-1.

Consistent with their reduced adhesion to VCAM-1, the α_4_ and β_1_ subunits of VLA-4 were reduced in both CD8 and CD4 T lymphocytes from four of five FAP patients tested (Fig. 2, D and E). The relative expression of both proteins, calculated as the ratio of their median fluorescence intensity in FAP versus matched control cells, was significantly lower in the former (Fig. 2E). One patient (FAP05) showed no reduction in α_4_ compared to its matched healthy donor, yet it had a lower expression of the β_1_ subunit, which would reduce the number of integrin complexes and may affect the adhesion in flow chambers (Fig. 2, A and B).

Therefore, T cells from FAP patients present impaired VLA-4 integrin–mediated adhesion to VCAM-1, also displaying reduced expression of VLA-4, potentially accounting for adhesion defects.

### APC silencing affects migration and adhesion in CEM T cells

**Fig. 3.**
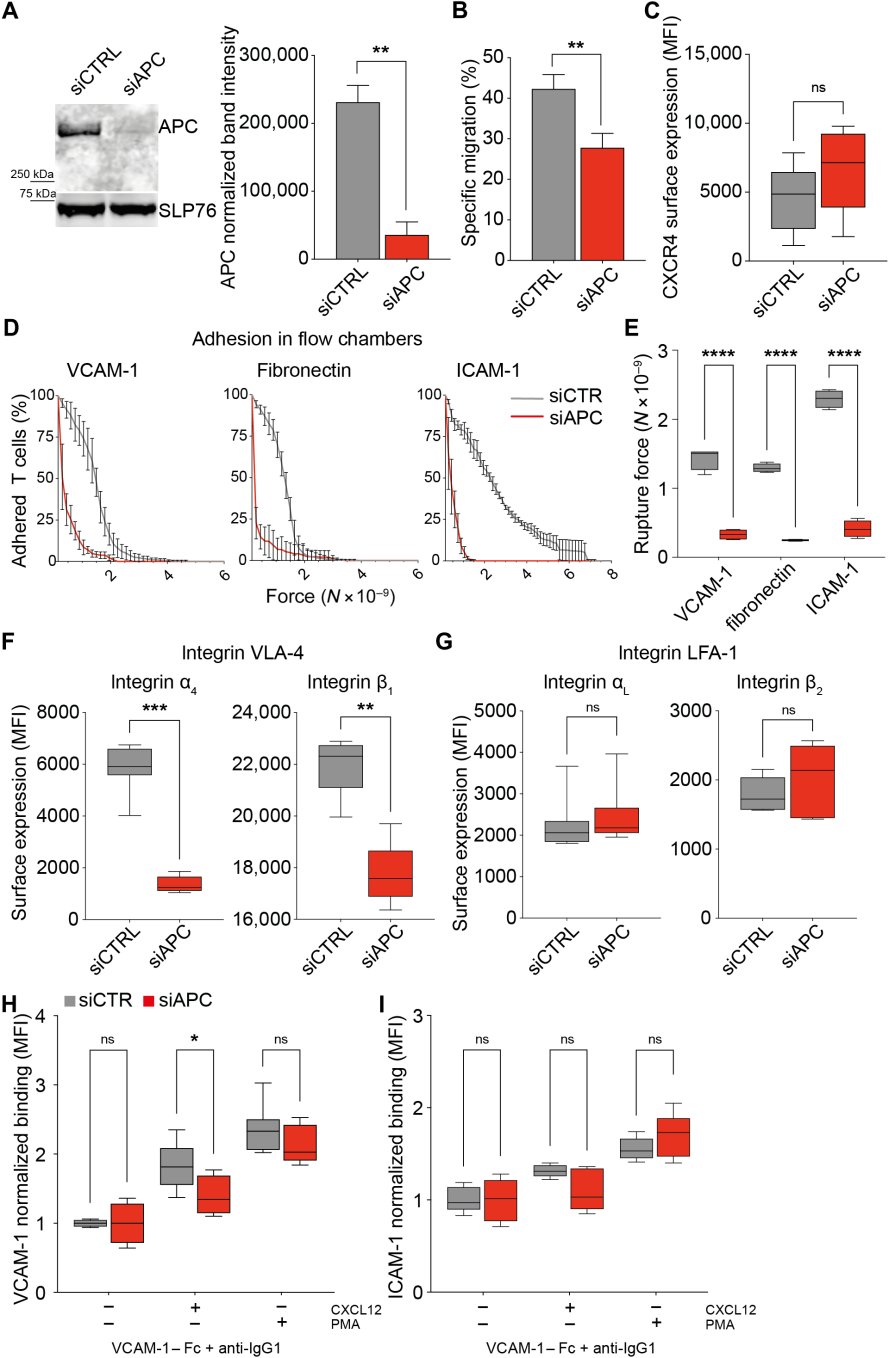
APC silencing impairs migration and adhesion of CEM T cells. CEM T cells were transfected with control (siCTRL) or APC (siAPC) siRNA oligonucleotides and used 72 hours after transfection. (**A**) Western blot showing the expression level of APC protein in siCTRL- and siAPC-transfected CEM T cells. A representative result is shown in the left panel. APC band intensity was quantified from four independent immunoblots and normalized by the SLP76 intensity in the same sample (right panel). (**B**) Transmigration through transwell filters was analyzed for control and APC-silenced CEM T cells. Bar plots represent the mean + SD of T cell–specific migration toward CXCL12. (**C**, **F**, and **G**) Surface expression of the CXCL12 chemokine receptor CXCR4 (C) and the α_4_β_1_/VLA-4 (F) and α_L_β_2_/LFA-1 (G) integrins was measured by flow cytometry. Boxes display the median fluorescence intensity. (**D** and **E**) siCTRL- and siAPC-CEM T cells were seeded in VCAM-1 or fibronectin or ICAM-1 + CXCL12–coated chambers, and a laminar shear flow of PBS was applied through the chamber (movie S3). (D) The number of adherent cells per frame was plotted as a function of the force on the different substrates. (E) Boxes represent the rupture force of adhesion to VCAM-1–, fibronectin-, and ICAM-1 + CXCL12–coated surfaces of siCTRL versus siAPC T cells. (**H** and **I**) VLA-4 and LFA-1 integrin activation was assessed by measuring the binding of T cells with the corresponding integrin ligands: VCAM-1 (H) and ICAM-1 (I). Boxes display the median fluorescence intensity. (A to I) Statistical differences (more than four independent experiments) were calculated by Mann-Whitney unpaired test. ****P < 0.0001, ***P < 0.001, **P < 0.01, and *P < 0.05; ns, not significant.

FAP patients are heterozygous and carry a variety of APC mutations that may result in null or truncated APC protein expression of the mutated allele and normal expression of the wild-type allele. Moreover, the number of cells obtained from patients is limited, precluding certain types of experiments. To generate an experimental system with a more homogeneous and controlled APC defect, providing enough cells to analyze the underlying molecular and cellular mechanisms, we investigated the effects of silencing APC expression by small interfering RNA (siRNA) in CEM tumor T cells (Fig. 3A) (*22*).

In line with the results from FAP patients (Figs. 1A and 2, A and B), we found that the CEM T cell ability to migrate through transwell filters in response to CXCL12 was impaired by APC silencing (Fig. 3B), despite no significant differences in CXCR4 expression (Fig. 3C). Likewise, their adhesion to VCAM-1 + CXCL12–coated microchambers under shear flow was reduced (Fig. 3, D and E, and movie S3). Moreover, adhesion to fibronectin and LFA-1 + CXCL12–coated surfaces was reduced (Fig. 3, D and E). While VLA-4 (α_4_, β_1_) expression was significantly decreased, LFA-1 (α_L_, β_2_) was not affected (Fig. 3, F and G). Last, we analyzed VLA-4 and LFA-1 activation by measuring binding of soluble recombinant VCAM-1 and ICAM-1, respectively. APC silencing impaired the increase of VCAM-1 binding in response to CXCL12. No significant changes on ICAM-1 binding were detected, although an inhibition trend was observed in CXCL12-stimulated Cells (Fig. 3, H and I).

Therefore, while reduced VLA-4 cell surface expression and activation may contribute to adhesion defects on VCAM-1 and fibronectin in APC-silenced cells, LFA-1 expression and activation may not explain the inhibition of adhesion to ICAM-1 + CXCL12–coated surfaces.

### APC-silenced CEM T cells display altered substrate contact patterns and activated integrin distribution

**Fig. 4.**
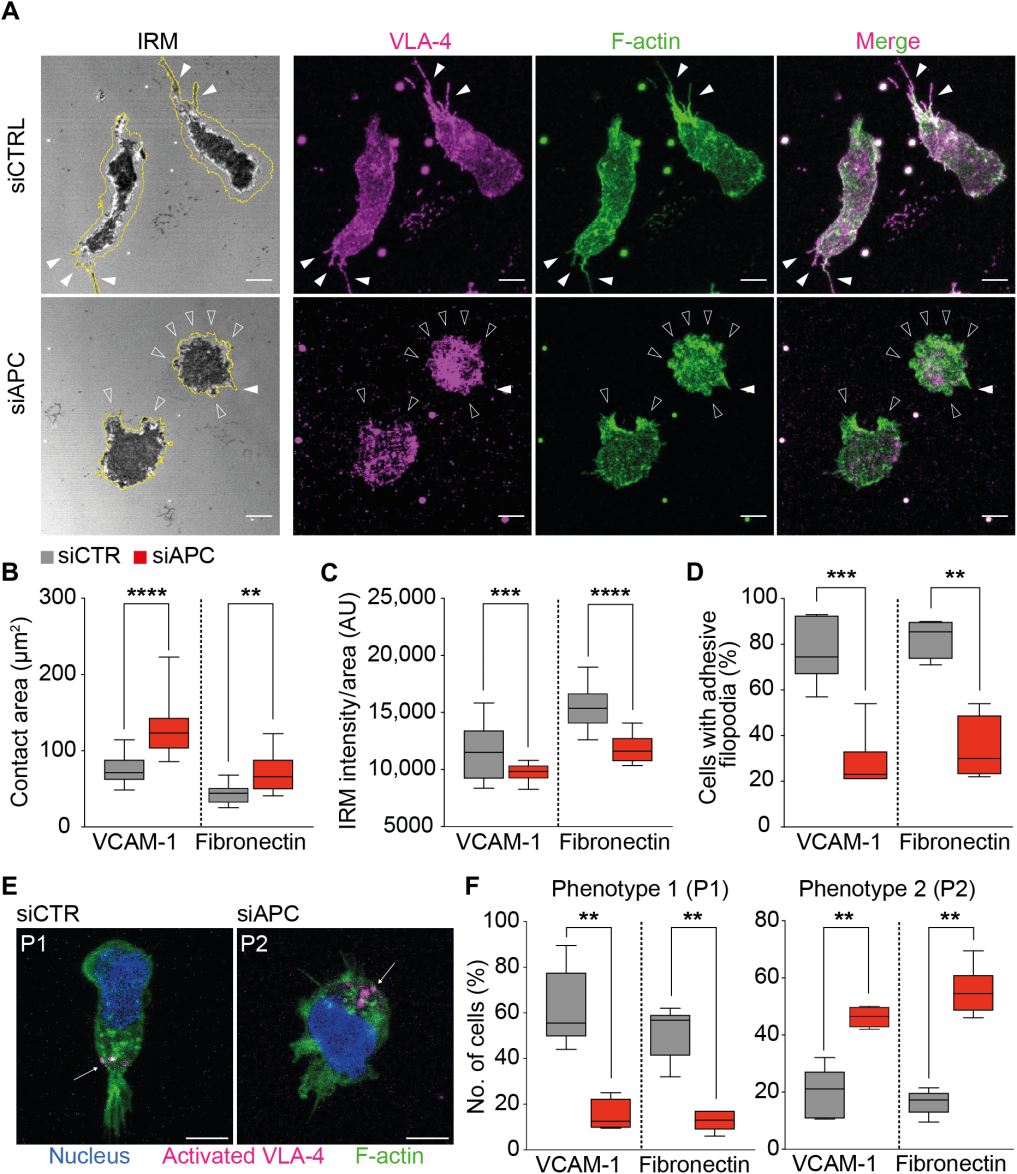
APC silencing alters VLA-4–dependent cell adhesion patterns. (**A** to **F**) CEM T cells were transfected with control (siCTRL) or APC (siAPC) siRNA oligonucleotides. Three days later, cells were seeded on VCAM-1– or fibronectin + CXCL12–coated surfaces and allowed to adhere and migrate for 10 min. Nonadherent cells were washed out, and dishes were fixed and stained with the indicated antibodies. T cell adhesive contacts of representative siCTRL- and siAPC-transfected cells analyzed by IRM and fluorescence confocal microscopy are shown (63× objective). Scale bars, 5 μm. (B to D and F) Boxes display measurements from at least two independent experiments (*n* > 100). Statistical differences were calculated by Mann-Whitney unpaired test. ****P < 0.0001, ***P < 0.001, and **P < 0.01. (A) Cells were segmented by thresholding on the F-actin channel to define their outline (yellow line). Darkest areas in the IRM image correspond to areas where the cell attaches the substratum and reveals a stronger adhesion. Filled arrowheads point at lateral and back thin protrusions contacting the substratum and enriched in VLA-4 and F-actin. Empty arrowheads indicate lobe-shaped protrusions typical of siAPC T cells. (B) T cell contact area with the substrate (in μm^2^) and (C) total pixel intensity of the contact area [in arbitrary units (AU)] were calculated from IRM images. (D) Percentage of cells displaying VLA-4–enriched filopodia was measured on fluorescence images. (E and F) HUTS-4 cluster appearance and distribution (see arrowheads) in migrating cells were classified by three independent investigators in two categories: phenotype 1 (P1), presence of small clusters segregated at the uropod, or phenotype 2 (P2), bigger clusters randomly positioned.

To further characterize the effect of APC silencing in impaired adhesion and migration, we analyzed the interaction of migrating cells with VCAM-1 or fibronectin + CXCL12–coated surfaces, imaging the area of cell-substrate contact by interference reflection microscopy (IRM) (*14*, *23*). APC silencing changed cell shape, resulting in increased contact area but weaker engagement with the surface, as shown by lower IRM intensity per cell (Fig. 4, A to C), in line with the reduced adhesion to VCAM-1 and the lower VLA-4 integrin expression (Fig. 3, D and F). Furthermore, APC-silenced T cells more often displayed rounder contact shapes (Fig. 4A) with lobe-shaped protrusions (Fig. 4A, empty arrowheads), while control cells were more elongated and displayed long, thin, IRM/F-actin/VLA-4–positive adhesive filopodia (Fig. 4, A, arrowheads, and D).

Last, we investigated the pattern of activated VLA-4 distribution, as assessed by staining with HUTS-4 antibody that detects a conformational change in the β_1_ subunit. Different HUTS-4 patterns were observed. While control cells displayed HUTS-4–positive puncta and small clusters in the uropod area, APC-silenced cells displayed larger and fewer clusters located anywhere in the unpolarized cell (Fig. 4, E and F). These differences may be due to distinct integrin anchoring to the cortical cytoskeleton potentially affected by APC silencing (see below).

These data add evidence that APC plays a crucial role in regulating adhesion and cytoskeleton coordination, necessary for lymphocytes to properly interact and migrate on adhesive substrates.

### APC-silenced CEM T cells have altered polarization and migrate extending and retracting unstructured lobes

**Fig. 5.**
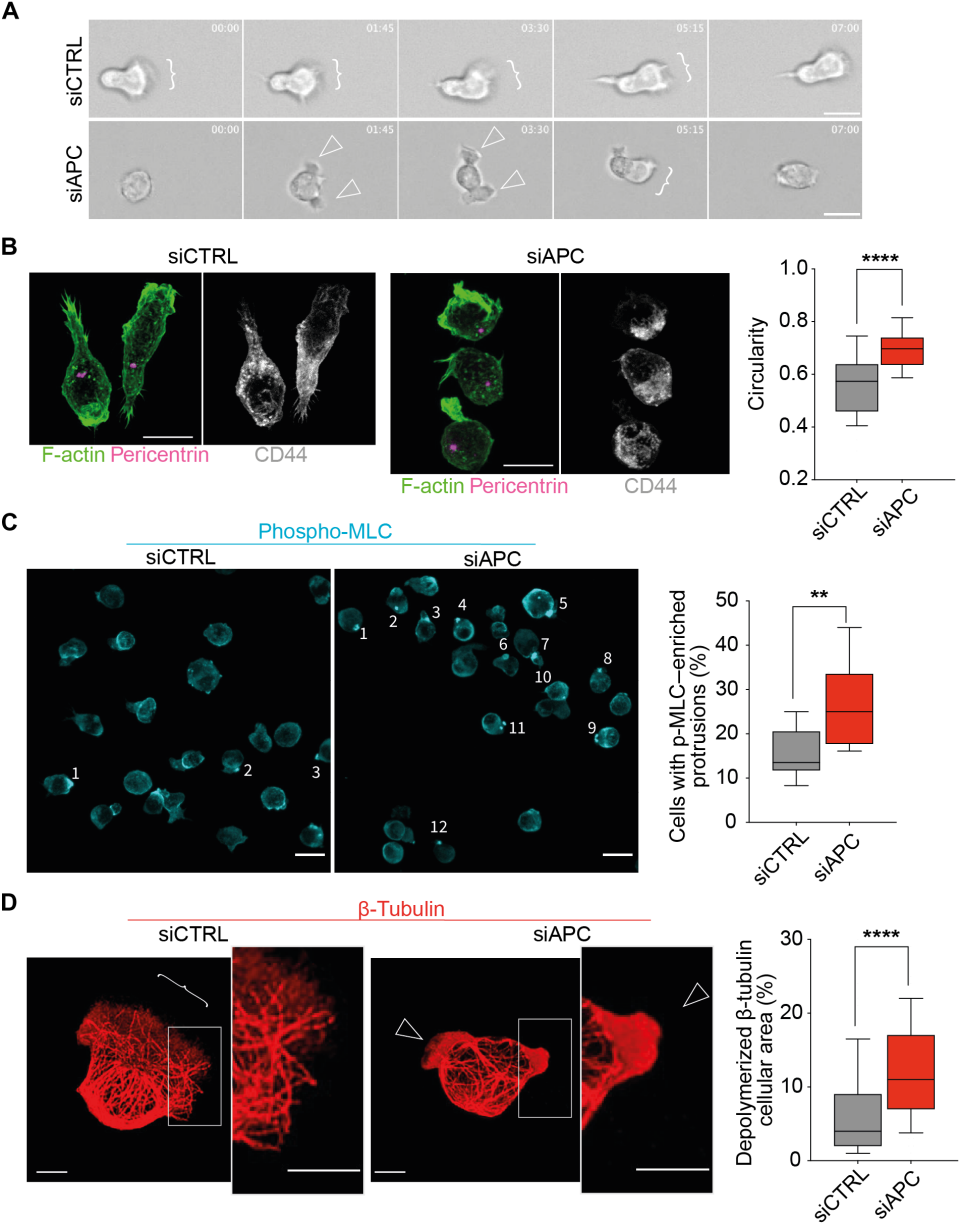
APC silencing modifies T cell dynamics, polarization, and cytoskeleton rearrangements. CEM T cells were transfected with control (siCTRL) or APC (siAPC) siRNA oligonucleotides and used after 72 hours. Cells were seeded on VCAM-1 + CXCL12–coated glass dishes (**A**) or coverslips (**B** to **D**) and allowed to adhere and migrate for 10 min. Thereafter, nonadherent cells were washed out. (A) The dynamics of migrating cells was analyzed by live-cell microcopy. Sequential frames of representative control and APC-silenced migrating T cell are shown (movie S4). Brackets indicate cell lamellipodia; empty arrowheads point at pseudopodia (20× objective). Scale bars, 10 μm. (B to D) Migrating cells were fixed and stained using phalloidin, anti-pericentrin, and anti-CD44 (B), anti-pMLC (C), or anti–β-tubulin (D) antibodies (63× objective). Scale bars, 5 μm. Statistical differences were calculated in three independent experiments (*n* > 100) by Mann-Whitney unpaired test. **P < 0.01 and ****P < 0.0001. (B) T cell polarization was assessed by analyzing the segregation of the polarity markers depicted in the confocal microscopy image. Circularity was measured with the following formula: circularity = 4π*area/perimeter^2^. A circularity value of 1.0 indicates a perfect circle. As the value approaches 0.0, it indicates an increasingly elongated shape. (C) Numbered cells in immunofluorescence images show accumulation of pMLC in small membrane protrusions. Scale bars, 5 μm. (D) Immunofluorescence images show microtubule network in siCTRL and siAPC CEM T cells. Insets show enlarged images of the framed areas corresponding to a cell lamellipodium (bracket) or a membrane extension containing depolymerized β-tubulin (empty arrowheads).

In agreement with the phenotype highlighted by IRM analyses in Fig. 4, imaging live-cell dynamics on the same surface showed APC-silenced CEM T cells often displaying several pseudopodia and round protrusions (Fig. 5A, empty arrowheads), whereas control cells displayed rather elongated shapes, with an evident front edge led by a dominant lamellipodium (Fig. 5A, brackets, and movie S4).

This polarized morphology adopted by control T cell while migrating was also accompanied by a specialized molecular segregation of T lymphocyte polarity markers. In agreement with previous reports (*13*), control cells displayed CD44 segregated at the uropod, the centrosome was localized behind the nucleus toward the back, and F-actin was concentrated at the front edge lamellipodium (Fig. 5B). In contrast to this molecular polarization, although in line with the pseudopodia-driven dynamics, APC-silenced cells displayed rounder shapes with several F-actin–rich pseudopodia and no properly defined front edge.

The motility behavior of APC-silenced T cells characterized by consecutive extension and retraction of pseudopodia prompted us to test whether these events may be acto-myosin–driven, as previously reported (*24*). In this line, we observed phosphorylated myosin light chain (pMLC)–enriched small protrusions more often present in APC-silenced T cells (Fig. 5C).

Last, more precise analysis of membrane extensions showed that APC-silenced T cells had more frequent protrusions containing depolymerized tubulin (Fig. 5D, empty arrowheads and zoomed area), instead of radial microtubules pointing to the front edge as in control cells (Fig. 5D, brackets and zoomed area). Therefore, APC silencing affects microtubule cytoskeleton, destabilizing lamellipodium areas and favoring lobe-type membrane extensions.

### APC silencing correlates with reduced ERM phosphorylation and T cell cortical rigidity

**Fig. 6.**
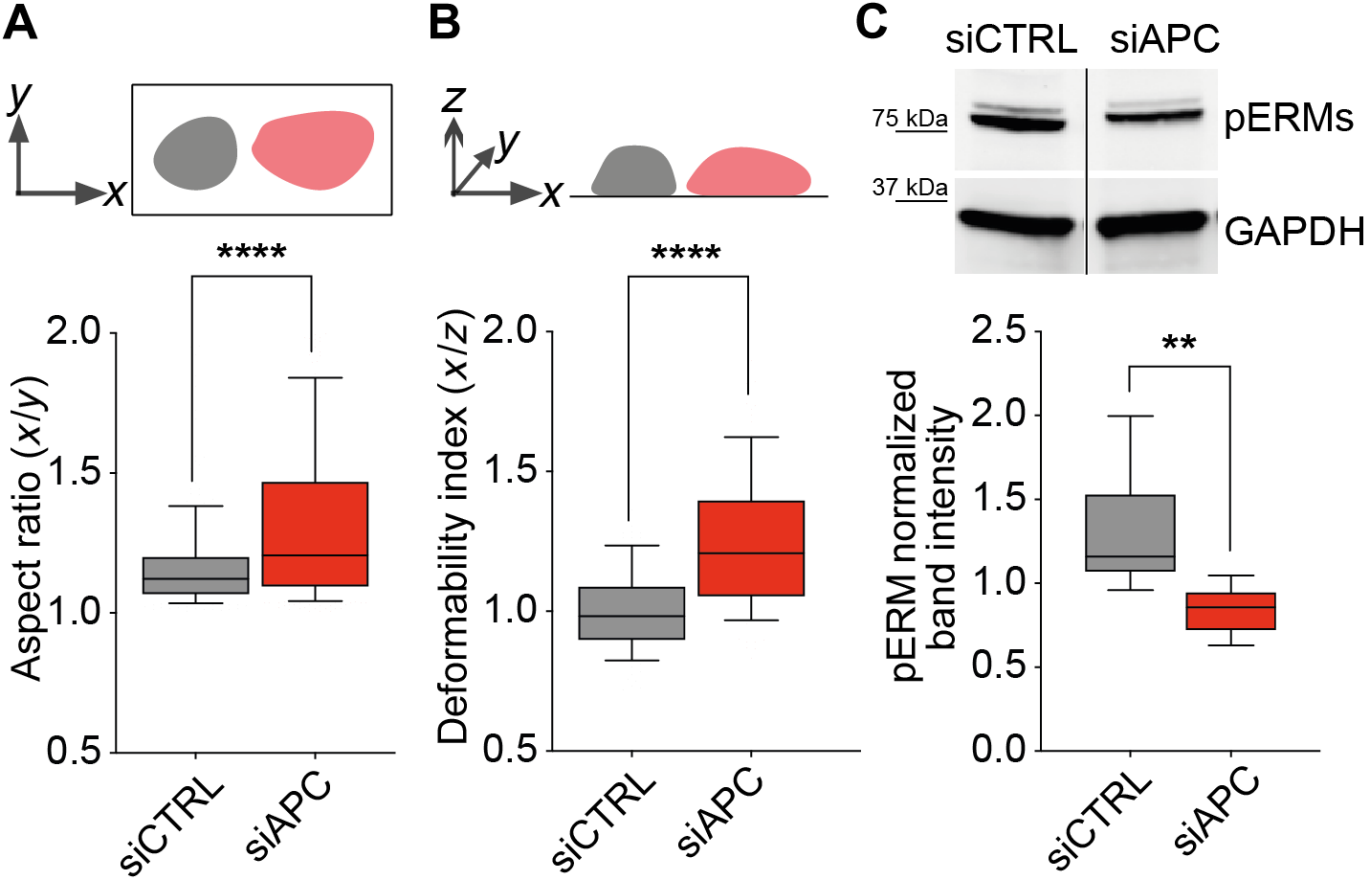
APC silencing alters cortical rigidity and ERM phosphorylation. CEM T cells were transfected with control (siCTRL) or APC (siAPC) siRNA oligonucleotides and used 72 hours after for the following analyses. (**A** and **B**) CellTrace Far Red dye–labeled cells were settled on poly-l-lysine–coated coverslips. After adding a PFA solution, cells were centrifuged at 3724*g* for 10 min. Cell deformation was quantified for each cell by measuring the aspect ratio (A) and the deformability index (B) and represented as box plots. Statistical differences were calculated by Mann-Whitney unpaired test. ****P < 0.0001. Aspect ratio and deformability indexes were calculated as follows: major axis (*x*)/minor axis (*y*) and major axis (*x*)/thickness (*z*), respectively. The more values deviate from 1, the more elongated or deformed cell shapes are. (**C**) Phospho-ezrin, Thr^567^, higher band and phospho-moesin, Thr^558^, lower band and glyceraldehyde-3-phosphate dehydrogenase (GAPDH) levels were assessed by Western blotting in siCTRL- and siAPC-transfected CEM T cells. ERM phosphorylation was quantified by near-fluorescence intensity and normalized to GAPDH intensity in the same sample. A representative immunoblot is shown in the left panel. Lanes were run on the same gel but were noncontiguous. Data are mean + SEM of four independent experiments. Statistical differences were calculated by Mann-Whitney test. **P < 0.01.

To explore the molecular bases of increased bleb-type protrusions in APC-silenced cells, we investigated whether ERM (ezrin-radixin-moesin) phosphorylation and cortical rigidity were affected by APC silencing. APC, through its interaction with Dlg1, may be part of a complex with ERMs that controls microtubule network organization at the T cell cortex (*6*, *25*). Moreover, ERMs, through their Thr phosphorylation, control the interactions between plasma membrane and the cortical actin cytoskeleton, thus regulating cortical rigidity and bleb generation (*26*). In addition, ERMs control Rho signaling and may therefore regulate local myosin-mediated contractility and keep the balance between lamellipodial versus bleb-type protrusions (*27*, *28*). Hence, we evaluated cell deformability under centrifugal force and ERM phosphorylation (*29*, *30*). APC-silenced cells showed a significantly higher tendency to flatten than control cells, as assessed by their increased aspect ratio and deformability index (Fig. 6, A and B). Moreover, APC-silenced cells displayed lower levels of pERMs (Fig. 6C), which may result in impaired cell cortex organization and stability (*26*, *30*).

Therefore, APC silencing may alter the organization of actin and microtubule cytoskeleton, leading to reduced cortical rigidity. Consequently, APC-silenced T cells appear more prone to form several membrane extensions instead of a dominant lamellipodium.

## DISCUSSION

Migration is a crucial step for immune cell functions, including T cell antitumor responses. We show here that the tumor suppressor and cell polarity regulator APC is key for T cell adhesion and migration. Our data reveal that T cells from FAP patients carrying APC mutations and APC-silenced CEM T cells display impaired migration. We identified integrin-mediated cell adhesion and cytoskeleton organization and stability as features targeted by APC defects. These are key requirements for T cells to migrate through physiological environments, as endothelium and lymphoid organs, or tumor tissues (*31*, *32*).

Cells migrate as the result of the interplay between physical constraints (e.g., stiffness, porosity, and order), substrate interactions (e.g., integrin-mediated adhesion), and chemical signals (e.g., chemokines) from the local microenvironment. These are mechanically integrated into membrane- and cytoskeleton-mediated cellular processes, such as adhesion, traction, protrusion, deformation, and polarization (*11*, *33*).

Using calibrated tools for in vitro cell migration, we identified APC as key for chemokine-induced T cell migration through micropores and HUVEC monolayers, and for spontaneous migration through narrow adhesive microchannels. Patients’ T cells showed impaired migration when migrating within microchannels coated with fibronectin, an extracellular matrix VLA-4 ligand. In contrast, on collagen, whose binding is VLA-4 independent, FAP patients’ T cells migrated faster than those from healthy subjects. Similarly, migration in 3D collagen matrices was not impaired in T cells from FAP patients. This is consistent with the low expression of α_1_ and α_2_ integrin subunits that, together with β_1_, form the collagen binding integrins. Therefore, APC mutations result in impaired VLA-4–dependent migration in confined environments.

In line with these findings, FAP patients’ T cells displayed lower adhesion force on surfaces coated with the VLA-4 integrin ligand VCAM-1 and the chemokine CXCL12, whose signaling reinforces integrin-mediated adhesion. Most of the FAP patients displayed a reduced VLA-4 cell surface expression in ex vivo activated T cells. Although the molecular mechanism linking APC defects to integrin expression impairment is unknown, this could be, at least in part, a cause of defective adhesion in patients’ T cells.

APC mutations in FAP patients may result in different functional phenotypes. Moreover, being a rare disease, the number and volume of samples were limited and precluded some mechanistic analyses. Therefore, to specifically target APC, enhance the phenotype, and gain access to larger number of cells, we investigated the effect of APC silencing in CEM leukemia T cells, a suitable model for T cell migration analyses (*22*). APC-silenced CEM T cells phenocopied migration, adhesion, and VLA-4 expression defects observed in FAP patient’s T cells, supporting the APC involvement in these functions. Accordingly, APC-silenced cells displayed impaired adhesion to surface coated with CXCL12 + VLA-4 ligands, i.e., VCAM-1 or fibronectin. Moreover, adhesion to CXCL12 + ICAM-1, the ligand for LFA-1, was also impaired. While VLA-4 expression and activation were affected in APC-silenced cells, LFA-1 expression or activation was not significantly altered. This suggests that APC differentially regulates VLA-4 and LFA-1 and that cytoskeleton disorganization induced by APC silencing may predominate in the adhesion impairment effect on ICAM-1.

To gain insight into the molecular and cellular processes involved, we used a 2D migration setup, where cells were allowed to migrate on VCAM-1 or fibronectin- and CXCL12-coated surfaces and observed by live-cell imaging or fixed, stained, and analyzed by confocal microscopy. Control cells adopted elongated shapes with distinguishable uropod and front lamellipodium, and frequent F-actin/VLA-4–enriched back filopodia, while APC-silenced cells were rounder and with fewer or no filopodia. These cells were also enriched in bigger, nonpolarized clusters of active VLA-4, further suggesting functional alterations of this integrin. In addition, they rarely polarized, but rather, they extended and retracted multiple pseudopodia on different directions, possibly affecting their migration persistence (*34*). Analyses of additional polarization markers, as ezrin and CD44 at the uropod and F-actin at the front edge, further supported that APC-silenced cells displayed impaired polarization. Generation of multiple pseudopodia was reminiscent of migration defects in T cells overexpressing a protein kinase Cζ (PKCζ) kinase-dead mutant (*35*). Cdc42 and Par6-PKCζ regulate the association of Dlg1 and APC to control cell polarization in migrating astrocytes (*36*).

Consistent with adhesion force differences, IRM revealed that control cell contacts with the substrate were tighter, although covering a smaller surface. APC-silenced T cells displayed also multiple small protrusions enriched in phosphorylated myosin and pseudopodia displaying F-actin but lacking structured microtubules. Since microtubule disorganization may trigger acto-myosin–driven retraction, both observations could be related (*37*, *38*). In contrast, in control cells, microtubules appeared structured, reaching lamellipodial edges, and pMLC-enriched protrusions were less frequent. This may facilitate sustained front edge advancing (*39*).

APC is part of the same polarity complex as Dlg1 that interacts with ezrin and moesin, which, in turn, control T cell cortex rigidity (*25*, *30*, *40*, *41*). We found that APC silencing reduced T cell cortical rigidity and steady-state phosphorylation of ezrin and moesin, suggesting cytoskeleton relaxation in APC-silenced T cells (*30*). This could also influence adhesion by altering integrin-cytoskeleton anchoring properties that control integrin avidity (*42*, *43*). Last, because of looser membrane interaction with the cortical cytoskeleton in APC-deficient cells, contraction forces and the consequent increase in hydrostatic pressure may favor membrane extensions and an ameboid-like migration on adhesive substrates (*26*, *44*). MLC phosphorylation and ERM proteins have been proposed to drive bleb-based motility of sphingosine-1-phosphate–stimulated T cells (*44*). Our findings suggest that similar mechanisms and molecular actors may also regulate bleb-type extensions of FAP patients’ T cells in fibronectin-coated microchannels. Bleb-mediated migration has been reported to preferentially occur under certain physically confined conditions (*26*, *45*).

In conclusion, our data provide the first evidence that APC orchestrates the coupling of cytoskeletal-mediated forces and integrin-dependent adhesion necessary for T cell migration as physiologically occurs across endothelial barriers, lymphoid organs, inflamed tissue and tumors. Consequently, APC defects may impair immune surveillance and effector functions of T cells, thus favoring tumorigenesis initiated by defects in epithelial cell differentiation in FAP patients.

## MATERIALS AND METHODS

### Patients

Seven FAP patients and their respective sex-, age-, and ethnicity-matched healthy donors were recruited through the Association Polyposes Familiales France and the Institut Pasteur ICAReB core facility [NSF 96-900 certified, from sampling to distribution, reference BB-0033-00062/ICAReB platform/Institut Pasteur, Paris, France/BBMRI AO203/1 distribution/access: 19 May 2016 (BIORESOURCE)], under CoSImmGEn protocols approved by the Committee of Protection of Persons, Ile de France-1 (no. 2010-dec-12483 for healthy donors and no. 2018-mai-14852 for FAP patients). Informed consent was obtained from all donors. Subjects were four women and three men. Average age was 48.8 years for healthy donors (range, 29 to 63) and 47.7 years for patients (29 to 60). Age matching was within a 0- to 5-year difference (average, 2.6). APC mutations included frameshift mutations potentially leading to lack of protein expression of the mutated allele or expression of truncated forms.

### Cells and transfections

Peripheral blood mononuclear cells were purified from FAP patients or healthy donors by Ficoll-Hypaque centrifugation and activated with TransAct (Miltenyi Biotec 130-111-160) (1:100) and recombinant human interleukin-2 (100 U/ml; PeproTech) in RPMI 1640 medium supplemented with GlutaMAX-I (Gibco), 5% human serum, 1 mM sodium pyruvate, nonessential amino acids, 10 mM Hepes, and 1% penicillin-streptomycin (v/v). Cell counts, viability and size were assessed every 2 days (up to day 12) by a LUNA-FL Dual Fluorescence Cell Counter, after cell staining with acridine orange/propidium iodide mixture (Logos Biosystems). Cell concentration was therefore adjusted for keeping cells at 2 × 10^6^/ml.

At day 5, CD4 T cells were purified using positive magnetic sorting (Miltenyi Biotec, 130-045-101) and CD8 T cells recovered from the nonretained population. Aliquots of activated T cells were frozen and settled back in culture later on for further experiments.

CEM T cells [acute lymphoblastoid leukemia cell line; (*46*)] were cultured in RPMI 1640 medium supplemented with 10% fetal bovine serum (FBS) and 1% (v/v) penicillin-streptomycin.

For siRNA transfections, 1 nmol of nontargeting control (Dharmacon, no. D-001210-01) or APC-targeting (Dharmacon, no. D-003869-06) siRNA was used per 10^7^ CEM T cells. Sequences used were previously described (*15*, *16*). Two transfections (1400 V, 10 ms, three pulses) were performed at a 24-hour interval with the Neon Transfection System (Invitrogen). Cells were analyzed 72 hours after.

### Chemotaxis

Primary T cells or CEM T cells [1 × 10^6^ cells/ml in RPMI 1640 and 0.5% bovine serum albumin (BSA)] were deposited in the upper inserts of transwell wells (Nunc). For trans-endothelial chemotaxis, a HUVEC monolayer was previously grown on these inserts as follows. Confluent HUVECs were gently trypsinized and seeded (0.2 × 10^6^ in 300 μl of complete EBM2 medium) in the upper compartments of the transwell inserts, previously coated with serum at 37°C for 2 hours. The lower compartments containing the same medium were refreshed the following day. After 3 days of culture, the integrity of confluent HUVEC monolayer was assessed by microscopy observation (fig. S1A) and by measuring the permeability of the monolayer using fluorescein isothiocyanate (FITC)–dextran (Sigma-Aldrich, #FD20S) diffusion. For T cell trans-endothelial chemotaxis, the upper compartments were gently rinsed in warm 0.5% BSA RPMI medium before seeding T cell.

The lower chambers contained the same medium supplemented or not with different concentrations of recombinant CXCL12 (R&D Systems, 350-NS) (40 ng/ml for CEM T cells if not otherwise indicated). After 90 and 60 min of incubation for primary and CEM T cells, respectively, at 37°C in 5% CO_2_, inserts were removed and the same volume of migrated and input populations was analyzed by flow cytometry. The percentage of CD8/CD4 T cells was assessed for primary T cells.

### Migration in microchannels

Microchannel experiments were performed as described (*47*). Briefly, customized polydimethylsiloxane (PDMS) microchips were coated with fibronectin (10 μg/ml) (Sigma-Aldrich, F1141) or collagen type I (SureCoat-5057) for 1 hour at room temperature (RT) and then washed with phosphate-buffered saline (PBS). T cells were labeled with Hoechst (Thermo Fisher Scientific, 33342) (200 ng/ml) for 30 min at 37°C, washed twice, and loaded in the microchips. Cell migration at 37°C in 5% CO_2_ was recorded overnight at one image per minute with a Leica DMi8 video microscope [10×/0.40–numerical aperture (NA) phase objective] and analyzed using a custom software.

### Cell migration in 3D collagen matrices

T cell migration in 3D was evaluated in customized PDMS chambers filled with a collagen I (Corning, 354249) mix (4 mg/ml) containing 0.72 × 10^6^ cells/ml. Gels were allowed to polymerize for 20 min at 37°C in 5% CO_2_ and equilibrated in RPMI 1640/5% human serum. Cell migration at 37°C in 5% CO_2_ was recorded at two images per minute (10×/0.40-NA phase objective) and analyzed using a custom software.

### Rupture force in laminar flow chambers

Flow chambers (μ-slide, Ibidi) were coated with rhVCAM-1/Fc Chimera (R&D Systems, 862-VC) + recombinant CXCL12 (R&D Systems, 350-NS) (1 μg/ml and 100 ng/ml in PBS, respectively) for 3 hours at RT, rinsed, and equilibrated with RPMI 1640 for 10 min at 37°C and then loaded with T cells for 10 min at 37°C. A flow rate of PBS (0 to 50 ml/min at 37°C) was applied through the temperature-controlled chamber for 92 s using a computer-driven syringe pump (SP210iW, World Precision Instruments) synchronized with image acquisition (three images per second) using an inverted transmission microscope (Axio Observer D1; Zeiss, 10×/0.3-NA objective) and Micro-Manager software (*48*). Images were analyzed using Fiji software (*49*) and the Cell Counter plugin. Forces necessary for cells to detach were calculated by determining the flow rate at rupture as described (*16*).

### Fluorescence and IRM

Coverslips or glass-bottom microwell dishes (MatTek) were coated with rhVCAM-1/Fc Chimera (R&D Systems, 862-VC) + recombinant CXCL12 (R&D Systems, 350-NS) (1 μg/ml and 100 ng/ml in PBS, respectively) for 2 hours at 37°C. They were further washed and blocked for 30 min with PBS/1% BSA, CaCl_2_, and MgCl_2_. CEM T cells (10^5^), resuspended in adhesion medium (PBS/1% FBS, 0.9 mM CaCl_2_, and 0.5 mM MgCl_2_ supplemented with Hepes), were allowed to migrate on coverslips or dishes for 10 min at 37°C, 5% CO_2_. Nonadhering cells were washed out, and the remaining ones were fixed for 12 min in 4% paraformaldehyde (PFA) and blocked in PBS/1% BSA overnight at 4°C. For microtubule detection, coverslips were additionally incubated for 5 min at −20°C in methanol before blocking. Fixed samples were incubated for 1 hour at RT with PBS/1% BSA and 0.1% Triton X-100 containing primary antibodies (Table 1), gently washed in PBS/1% BSA, and incubated for 45 min at RT with the corresponding fluorochrome-coupled secondary antibodies and phalloidin (Table 1). Coverslips were mounted on microscope slides using ProLong Gold Antifade mounting medium with 4′,6-diamidino-2-phenylindole (DAPI) (Life Technologies).

**Table 1.** Primary and secondary antibodies/reagents used for immunofluorescence.

**Antibodies/reagents—clone**	**Host-isotype**	**Reference**	**Dilution**
Acti-stain 488 fluorescent phalloidin	Phallotoxin	Cytoskeleton PDHG1	1:50
Anti-human CD49D purified clone 9F10	Mouse IgG1	eBioscience 14-0499	1:75
Anti-human CD29 purified clone TS2/16	Mouse IgG1	eBioscience 14-0299	1:75
Anti–phospho-myosin light chain 2 (Ser^19^)	Mouse IgG1	Cell Signaling Technology 3675	1:100
Anti-pericentrin	Rabbit IgG	Abcam ab4448	1:500
Anti-CD44, clone G44-26	Mouse IgG2b	BD 550392	1:200
Anti-tubulin, beta, clone KMX1	Mouse IgG2b	Millipore MAB3408	1:500
Anti-mouse IgG2b, human ads-FITC	Goat	SouthernBiotech 1090-02	1:100
Cy3-conjugated AffiniPure anti-mouse IgG1	Goat	Jackson ImmunoResearch 115-165-205	1:100
Cy5-conjugated AffiniPure anti-mouse IgG1	Goat	Jackson ImmunoResearch 115-175-205	1:100
Anti-fluorescein/Oregon Green, Alexa Fluor 488 conjugate	Goat IgG1	Invitrogen A11096	1:100

Images were acquired with an LSM700 confocal microscope. For IRM, we used an LSM780 confocal microscope equipped with an 80/20 beam splitter and a variable bandwidth chromatic filter. Confocal optical sections (63×/1.40-NA objective) were acquired using ZEN software (Zeiss). Images were analyzed with Fiji software (*49*).

### Flow cytometry

T cell surface protein staining was carried out during 40 min at 4°C with appropriate conjugated fluorophore antibodies (Table 2) prepared in PBS/2% serum/0.05% NaN. A total of 100,000 events in the viability gate (Fixable Viability Stain 450, BD 562247) were acquired on the MACSQuant Analyzer (Miltenyi Biotec) and processed with Kaluza software.

**Table 2.** Antibodies used for flow cytometry.

**Antibodies—clone**	**Host-isotype**	**Reference**	**Dilution**
PE/Cy5 anti-human CD3 clone HIT3a	Mouse IgG2a	BD 555341	1:30
FITC anti-human CD4 clone RPA-T4	Mouse IgG1	BioLegend 300506	1:50
APC/Cy7 anti-human CD8a clone RPA-T8	Mouse IgG1	BioLegend 301016	1:30
PE anti-human CD49d clone MZ18-24A9	Mouse IgG2b	Miltenyi Biotec 130-093-282	1:50
PE anti-human CD49a clone TS2/7	Mouse IgG1	BioLegend 328304	1:50
APC anti-human CD49b clone P1E6-C5	Mouse IgG1	BioLegend 359310	1:50
APC anti-human CD11a	Mouse IgG1	Miltenyi Biotec 130-127-294	1:50
PE/Cy7 anti-human CD29 clone TS2/16	Mouse IgG1	BioLegend 303026	1:50
PE/Cy5 anti-human CD18 clone TS1/18	Mouse IgG1	BioLegend 302109	1:50
APC anti-human CXCR4 clone 12G5	Mouse IgG2a	Invitrogen 17-9999	1:75

### Analysis of VLA-4 and LFA-1 activation

To assess the integrin activation, we measured the ability of cells to bind the ligands for VLA-4 and LFA-1, i.e., VCAM-1 and ICAM-1, respectively (*50*). Briefly, recombinant human chimeric protein VCAM-1–IgG1 Fc (R&D Systems, 862-VC) or ICAM-1–IgG1 Fc (R&D Systems, 720-IC) was coupled with an anti–human IgG1 AF488-labeled antibody (Invitrogen, A10631). CEM T cells were resuspended at 3 × 10^6^ cells/ml in RPMI 1640, supplemented with 2% FBS, 1.8 mM CaCl_2_, and 1 mM MgCl_2_. Thereafter, they were incubated with a mix (5:1 volumes) containing VCAM-1 or ICAM-1 complexes and stimulating molecules, i.e., CXCL12 (R&D Systems, 350-NS; final concentration, 250 ng/ml) or phorbol 12-myristate 13-acetate (PMA; 50 ng/ml; Sigma-Aldrich, P8139). Cells were further fixed for 15 min in 4% PFA and then washed twice in PBS/2% serum/0.05% NaN. Samples were acquired on the MACSQuant Analyzer (Miltenyi Biotec), and data were processed with Kaluza software. Cell fluorescence intensity was used to assess the effective VCAM-1 or ICAM-1 binding to their respective ligands.

VLA-4 activation was also assessed in migrating CEM T cells. Briefly, siCTRL- or siAPC-transfected CEM T cells were allowed to migrate on VCAM-1– and CXCL12-coated surfaces as described above in the presence of the anti-activated integrin β_1_ antibody (clone HUTS-4; Sigma-Aldrich, #MAB2079-AF647, 1:100). Ten minutes later, nonadhering cells were washed out and the remaining ones were fixed in 4% PFA, permeabilized, and stained as indicated above (see the previous section).

### Random migration on adhesive surfaces

Glass-bottom microwell dishes (MatTek) were coated as described above. CEM T cells (10^5^) were resuspended in adhesion medium and seeded on dishes for 10 min at 37°C. Nonadhering cells were washed out, and the dish was placed in the thermostatic humid chamber of a Definite-Focus video microscope. Cell migration at 37°C in 5% CO_2_ atmosphere was recorded at five images per minute during 20 min (20×/0.40-NA objective) and analyzed using Fiji software (*49*).

### Cortical rigidity

The experimental procedure has been described (*29*, *30*). CEM T cells were labeled with 0.5 mM CellTrace Far Red DDAO-SE (Invitrogen C34553) and resuspended in RPMI 1640 without serum. Cells were settled on poly-l-lysine–coated coverslips and placed in 24-well plates. Same volume of 4% PFA was added to the wells, and plates were centrifuged for 10 min at 3724*g*. Coverslips were then mounted on slides using ProLong Gold Antifade mounting medium. Images were acquired with a LSM700 confocal microscope (Zeiss) (6×/1.40-NA objective) and analyzed using Fiji software (*49*).

For 2D morphological analyses, Fiji was used to build images (z-stack). Then, an autothresholding algorithm in Fiji was used to outline cells based on their shape and dimensions. Particles smaller than 7 μm were excluded. Aspect ratio was calculated for each cell as follows: major axis (*x*)/minor axis (*y*). As the value deviates from 1.0, it indicates an increasingly elongated shape.

For 3D morphological studies, Fiji was used to build *x*-*z* images (z-projection). The maximum *x* and the maximum *z* of each cell were measured by using the Fiji stick. Deformation index was calculated for each cell as follows: major axis (*x*)/thickness (*z*). As the value deviates from 1.0, it indicates an increasingly deformed shape.

### Western blot

For immunodetection of APC and phosphorylated proteins, 2 × 10^6^ and 1 × 10^6^ of CEM T cell lysates, respectively, were processed as previously described (*15*, *16*). Primary antibodies (Table 3) were incubated overnight at 4°C. Secondary antibodies conjugated with Alexa Fluor 680 or DyLight 800 (Thermo Fisher Scientific) were applied for 35 min at RT in the dark. Detection was performed using the Odyssey Classic Near-Infrared Imaging System (LI-COR-Biosciences).

**Table 3.** Antibodies used for Western blot.

**Antibodies—clone**	**Host-isotype**	**Reference**	**Dilution**
Anti-APC ALi 12-28	Mouse IgG1	Abcam ab58	1:500
Anti-SLP76	Rabbit IgG	Thermo Fisher Scientific PA5-17556	1:1000
Anti-phospho-ezrin (Thr^567^)/radixin (Thr^563^)/moesin (Thr^558^) clone 48G2	Rabbit IgG	Cell Signaling Technology 3726	1:1000
Anti-GAPDH clone 6C5	Mouse IgG	Calbiochem CB1001	1:1000

### Statistical analysis

Statistical analyses were carried out using GraphPad Prism V.9. Details are depicted in individual figure legends. The *P* values are represented as follows: ****P < 0.0001, ***P < 0.001, **P < 0.01, *P < 0.05, and ns *P* ≥ 0.05.

## Notes

View/request a protocol for this paper from *Bio-protocol*.
