# Supplementary material for: The tumor suppressor adenomatous polyposis coli regulates T lymphocyte migration
Source: Sci Adv. 2022 Apr 13;8(15):eabl5942. doi: 10.1126/sciadv.abl5942 (PMC9007504; doi:10.1126/sciadv.abl5942)
Supplement: Supplementary file 1 — Figs. S1 and S2 [file sciadv.abl5942_sm.pdf]

Science Advances
AAAS

Supplementary Materials for

The tumor suppressor adenomatous polyposis coli regulates T lymphocyte migration

Marta Mastrogiovanni, Pablo Vargas, Thierry Rose, Céline Cuche, Elric Esposito, Marie Juzans, Hélène Laude, Amandine Schneider, Mathilde Bernard, Sophie Goyard, Charlotte Renaudat, Marie-Noëlle Ungeheuer, Jérôme Delon, Andrés Alcover*, Vincenzo Di Bartolo*

*Corresponding author. Email: andres.alcover@pasteur.fr (A.A.); vincenzo.di-bartolo@pasteur.fr (V.D.B.)

Published 13 April 2022, *Sci. Adv.* **8**, eabl5942 (2022)
DOI: 10.1126/sciadv.abl5942

The PDF file includes:

Figs. S1 and S2
Legends for movies S1 to S4

Other Supplementary Material for this manuscript includes the following:

Movies S1 to S4

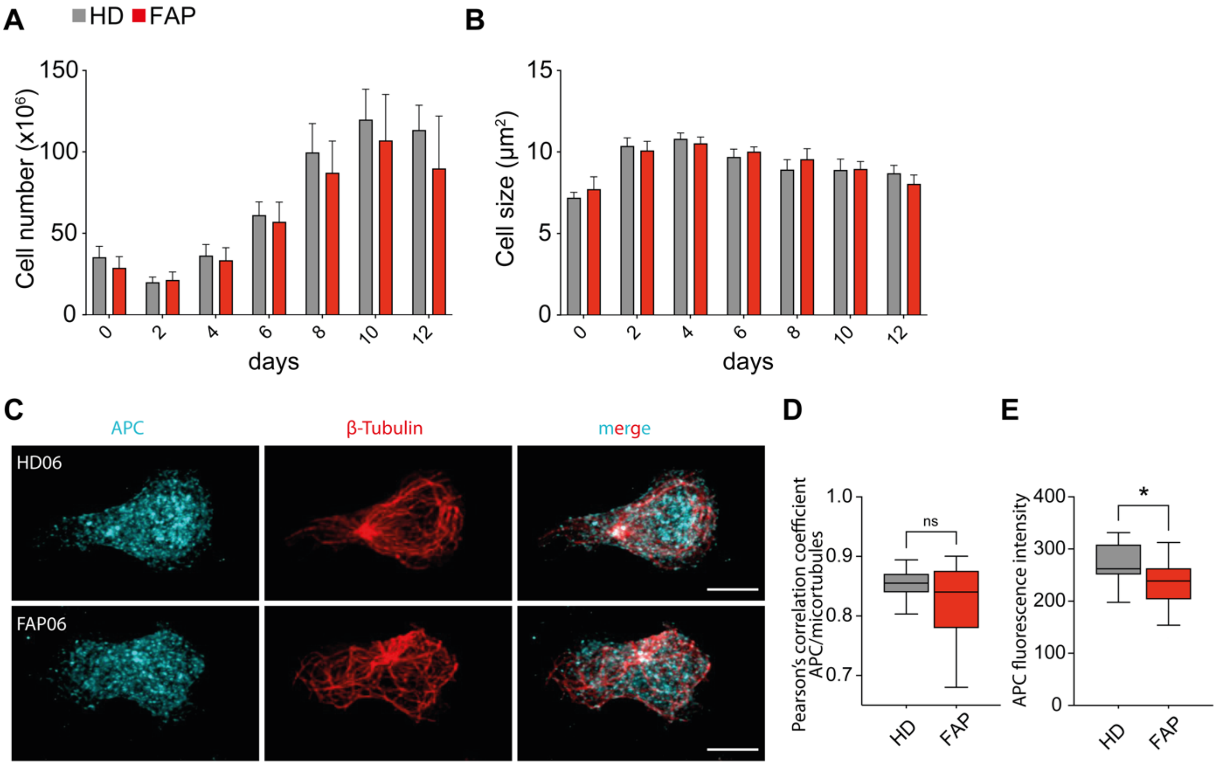

Fig. S1. Characterization of T lymphocytes from FAP patients and matched healthy individuals. PBMCs were purified from FAP patients and matched healthy donors. They were put in culture and stimulated as described in Methods. (**A,B**) Cell counts and size were assessed every two days for 12 days. Bars display mean + SEM of measurements from 7 individual pairs. Statistical differences were calculated by 2way ANOVA with the Sidak correction for multiple comparisons. Differences were not significant at any time point. (**C-E**) CD8 T cells were purified and their migration on VCAM-1 + CXCL12-coated surfaces was assessed. After 10 minutes, non-adherent cells were washed out and dishes were fixed and stained for the depicted antibodies. (**C**) CD8 T cells from a representative individual pair si shown. 63x objective. Scale bars 5 µm. (**D,E**) Images were processed using the Fiji software for measuring the APC-microtubule colocalization (**D**), with the Colocalization Threshold plugin giving the Pearson's coefficient, and the APC fluorescence intensity (**E**). Boxes display measurements from 3 individual pairs. Statistical differences were calculated by Mann-Whitney unpaired test. * $p < 0.05$, ns = not significant.

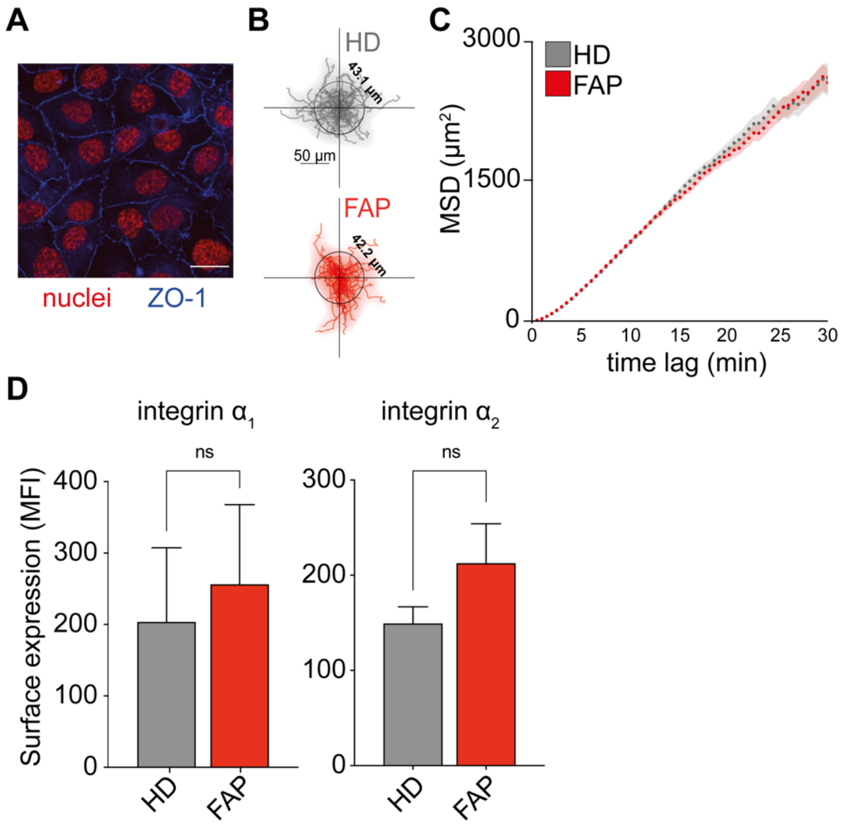

Fig. S2. Assessment of HUVEC cell layers for T cell trans-endothelial chemotaxis, analysis of migration in collagen matrices and of α integrins surface expression (Related to Figure 1B,C and 2D,E). (**A**) To assess trans-endothelial chemotaxis, HUVEC cells were seeded on serum-coated 5 µm-pore transwell filters. Three days later, the integrity of confluent HUVEC monolayer was analyzed by fluorescence microscopy. ZO-1 staining (blue) indicates mature tight junctions between HUVEC cells in the layer. Cell nuclei are stained in red. 63x objective. Scale bar 20 µm. (**B**) CD8 T cells were assessed for 3D migration in collagen gels. Trajectory plots of CD8 T cells migrating in 3D collagen gels are shown for one representative individual pair (FAP03) out of four. Migration was imaged during 30 minutes. The starting point of each trajectory was translated to the origin of the plot. (**C**) Mean squared displacement quantified from the data depicted in (**B**). (**D**) The surface expression of the α_1 and α_2 subunits of the collagen receptor was assessed by flow cytometry. Bar plots represent the mean + SEM of the integrin median fluorescence intensity from 3 individual pairs (FAP01-FAP03). Statistical differences were calculated by paired two-tailed t-test. ns = not significant.

Movie S1. FAP patients' CD8 T cells display impaired migration in fibronectin-coated microchannels (Related to Figure 1E-F). CD8 T cells from a HD or FAP were allowed to migrate in microchannels of 4μm width and 5μm height, previously coated with fibronectin. HD T cell migration (top panel) is faster and driven by a front dominant lamellipodium, whereas the FAP T cell one (bottom panel) is slower and characterized by the formation of blebs. This video is representative of 5 HD-FAP individual pairs. Time stamp is in hr:min:sec. 63x oil objective. Scale bar 10μm.

Movie S2. FAP patients' CD8 T cells detach at lower laminar flow forces in adhesion chambers (Related to Figure 2A-B). CD8 T cells from a HD or FAP are allowed to seed in a temperature-controlled flow chamber, previously coated with VCAM-1 + CXCL12. A linear flow rate ramp of PBS (37°C), increasing from 0 to 50 mL/min, is applied to the chamber for 92s using a syringe pump. FAP patients' T cells (right panel) detach at lower flow intensity than HD cells (left panel), indicating defects in their adhesion strength. This video is representative of 7 HD-FAP individual pairs. Time stamp is in min:sec. 10x/0.3 objective. Scale bar 50μm.

Movie S3. APC-silenced T cells detach at lower laminar flow forces in adhesion chambers (Related to Figure 3C) siRNA-transfected CEM T cells are allowed to seed in a temperature-controlled flow chamber, previously coated with VCAM-1 + CXCL12. Image acquisition starts when a linear flow rate ramp of PBS (37°C), increasing from 0 to 50 mL/min, is applied to the chamber for 92s using a syringe pump. siAPC T cells (right panel) detach at lower flow intensity than siCTR cells (left panel), indicating defects in their adhesion strength. This video is representative of 4 independent experiments. Time stamp is in min:sec. 10x/0.3 objective. Scale bar 50μm.

Movie S4. APC-silenced CEM T cells display a pseudopodia extension-retraction migration mode when migrating on adhesive substrates (Related to Figure 5A). siRNA-transfected CEM T cells are allowed to migrate on VCAM-1 + CXLC12-coated dishes. siCTR T cell (left panel) migration is leaded by the extension of a unique front lamellipodium. A thin extension is also visible at the uropod. The APC-silenced T cell migration pattern is rather characterized by the processive extension and retraction of membrane protrusions resulting in a not directional locomotion. This video is representative of 3 independent experiments. Time stamp is in min:sec. 20x/0.4 objective. Scale bar 10μm.
